# Bare Iron Oxide Nanoparticles as Drug Delivery Carrier for the Short Cationic Peptide Lasioglossin

**DOI:** 10.3390/ph14050405

**Published:** 2021-04-24

**Authors:** Chiara Turrina, Sonja Berensmeier, Sebastian P. Schwaminger

**Affiliations:** Bioseparation Engineering Group, Department of Mechanical Engineering, Technical University of Munich, 80333 München, Germany; c.turrina@tum.de (C.T.); s.berensmeier@tum.de (S.B.)

**Keywords:** iron oxide nanoparticles, magnetically controlled drug delivery, cationic peptide, lasioglossin, agglomeration behavior in human serum, antimicrobial behavior

## Abstract

New drug delivery systems are a potential solution for administering drugs to reduce common side effects of traditional methods, such as in cancer therapy. Iron oxide nanoparticles (IONs) can increase the drugs’ biological activity through high binding efficiency and magnetically targeted drug delivery. Understanding the adsorption and release process of a drug to the carrier material plays a significant role in research to generate an applicable and controlled drug delivery system. This contribution focuses on the binding patterns of the peptide lasioglossin III from bee venom on bare IONs. Lasioglossin has a high antimicrobial behavior and due to its cationic properties, it has high binding potential. Considering the influence of pH, the buffer type, the particle concentration, and time, the highest drug loading of 22.7% is achieved in phosphate-buffered saline. Analysis of the desorption conditions revealed temperature and salt concentration sensitivity. The nanoparticles and peptide-ION complexes are analyzed with dynamic light scattering, zeta potential, and infrared spectroscopy. Additionally, cytotoxicity experiments performed on *Escherichia coli* show higher antimicrobial activity of bound lasioglossin than of the free peptide. Therefore, bare IONs are an interesting platform material for the development of drug-delivery carriers for cationic peptides.

## 1. Introduction

**Figure 1:**
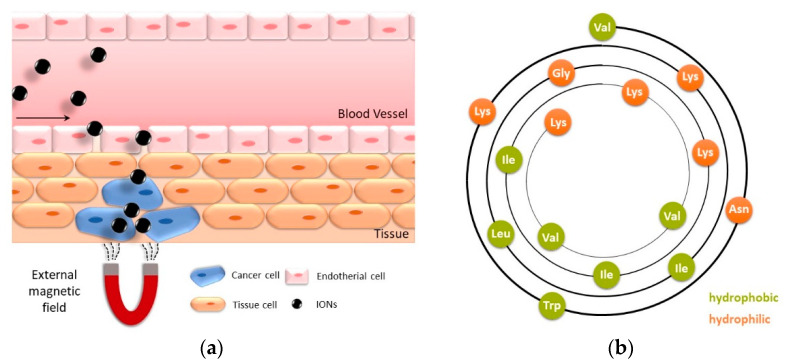
(**a**) Drug delivery of magnetic IONs accumulating in a target tissue, guided by an external magnetic field. (**b**) Wheel diagram of lasioglossin III: hydrophilic amino acids are shown in orange, and hydrophobic amino acids are shown in green.

The method of administering a pharmaceutical compound highly influences the therapeutic effect, efficiency, and drug safety [1,2]. Magnetically controlled drug delivery is attracting increasing attention due to the potential to carry a large drug dose to the target, which leads to a high local concentration and thereby high efficiency while avoiding toxicity [1,3,4,5,6] Z. Superparamagnetic iron oxide nanoparticles (IONs), also known as magnetite/maghemite or magnetic nanoparticles (MNP), are especially in the focus of this research field due to their non-remanent behavior, non-toxicity, and low-cost production [7,8,9,10,11]. IONs are non-porous and can have high specific surface areas of above 100 m^2^/g, leading to a huge drug loading capability [12]. The classic synthesis route is co-precipitation via the Massart process. This method can be used to generate IONs with a size range between 4 and 16 nm that have a high magnetization of around 80 emu/g [9,13,14,15]. The key points of IONs as a drug carrier are the possibility of targeted delivery through a magnetic field, the visualization of the delivery process via MRI, and that heat generated through hyperthermia can lead to a controlled release [16,17]. For the internalization of IONs into the cell, the ideal particle size for drug delivery ranges from 10 to 100 nm because this leads to the most prolonged blood circulation times [9,13,18]. The drug can be localized at the target site by applying an external magnetic field, where it can be efficiently released from its carrier, as shown in Figure 1a [15].

IONs are often coated with functionalized, biocompatible compounds to form a core–shell structure for biomedical application [9]. Therefore, drugs can bind to the coating or are dispersed in the polymer matrix [16,19]. The polymers have the function of stabilizing the particles, preventing early immunogenic action, or are used to generate a controlled release mechanism [15,18,20]. In this case, the interaction with the drug, the cationic peptide lasioglossin (LL), should occur with the bare surface of IONs without coating. In drug delivery or bioseparation, the controlled adsorption and release of small biomolecules to and from the adsorbent material plays a major role. During the last few years, much research has been conducted to understand small biomolecules’ interaction with the inorganic bare IONs (BIONs) and amphoteric hydroxyl groups on the metal oxide surface [21,22]. The surface charge influences the adsorption behavior of amino acids as well as the amount of charged groups such as carboxylic acids and side chains. The coordinative complex formation due to ionic interactions of the iron ions on the surface of BIONs is pH-dependent [22,23]. Rawlings et al. have proven that lysine can undergo strong hydrogen bonds with the surface of BIONs through the amine group and the peptide carbonyl [24]. This makes LLs an interesting counterpart for adsorption on BIONs.

LLs are cationic, α-helical pentadecapeptides isolated from bee venom of *Lasioglossum lacticeps.* They belong to the group of antimicrobial peptides that are a new alternative for antibiotics, based on their divergent mode of action. The ability of LL to form an amphipathic α-helical structure highly influences their biological activity [25,26,27,28]. Cationic peptides can target the negatively charged bacterial cell envelope and accumulate in the cell wall. This leads to the formation of transmembrane pores into the lipid bilayer, the leakage of cytoplasmic components, and therefore to cell death [29,30]. LLs appear in three different natural forms (LL I: H-Val-Asn-Trp-Lys-Lys-Val-Leu-Gly-Lys-Ile-Ile-Lys-Val-Ala-Lys-NH2, LL II: H-Val-Asn-Trp-Lys-Lys-Ile-Leu-Gly-Lys-Ile-Ile-Lys-Val-Ala-Lys-NH2, LL III: H-Val-Asn-Trp-Lys-Lys-Ile-Leu-Gly-Lys-Ile-Ile-Lys-Val-Val-Lys-NH2). They all show low hemolytic and high antimicrobial behavior even at physiological salt concentrations [31]. LL III is shown in Figure 1b. The peptide is positively charged due to its five lysine residues, and it can undergo electrostatic interactions with a negatively charged BION surface [24]. The peptide has a concave hydrophobic and a convex hydrophilic side through the α-helical shape. All LLs show similar antimicrobial behavior against Gram-negative and Gram-positive bacteria such as *E. coli*, *P. aeruginosa,* and *B. subtilis* [25]. Out of the three natural peptides, LL III shows the highest activity in growth studies of *S. aureus* due to its highly hydrophobic behavior. While all LLs show potency to kill various types of cancer cells, LL III has the highest toxicity against PC-12 cancer cells [25]. 

This work aims to successfully generate and analyze BION@LL complexes. The particles are characterized in detail before their application in adsorption and desorption is described. The particle size and size distribution are important parameters regarding particle delivery through the human vascular system and removal through the organs and the immune system [16]. They are determined by transmission electron microscopy (TEM) and X-ray diffraction analysis (XRD). The diffractograms are further used for the study of the crystal structure that influences the magnetic behavior. The saturation magnetization and magnetic behavior are analyzed with a superconducting quantum interference device (SQUID). Additionally, the zeta potential is investigated in dependence of the pH to calculate the isoelectric point (IEP). The knowledge of the surface charge makes it possible to understand the interaction with LL in adsorption and desorption experiments. The peptide loading to BION’s surface is verified by photometric measurements and infrared spectroscopy (IR). The hydrodynamic diameter of the BION@LL complexes is analyzed with dynamic light scattering, as the size of the system plays a major role in the application in drug delivery [16]. The binding behavior of LL is tested at different pH values. Additionally, the influence of PBS buffer, particle concentration, and time are determined. For the elution process, other conditions such as a pH shift, rise of temperature, and variation of the salt concentration are analyzed. The antimicrobial behavior of LL, BIONs, and the BION@LL complex is compared to test the applicability in drug delivery. The aim is to generate a fast, controllable, efficient, and reversible LL interaction with the BIONs and high antimicrobial activity of the resulting magnetic complexes.

## 2. Results and Discussion

### 2.1. Characterization of the BIONs

**Figure 2:**
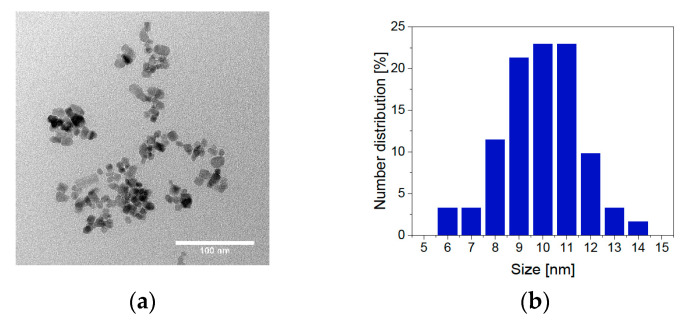
TEM microscopy: (**a**) image of BIONs and (**b**) number distribution of various particle diameters.

The BIONs are characterized by particle size, size distribution, crystal structure, BET surface, and magnetization behavior. All these parameters play an essential role in the application in magnetic drug delivery. They influence the particle lifetime in the human vascular system, the possibility of magnetic separation, and the amount of peptide bond to the surface. With TEM analysis, the optical diameter of the particles is examined (Figure 2).

**Figure 3:**
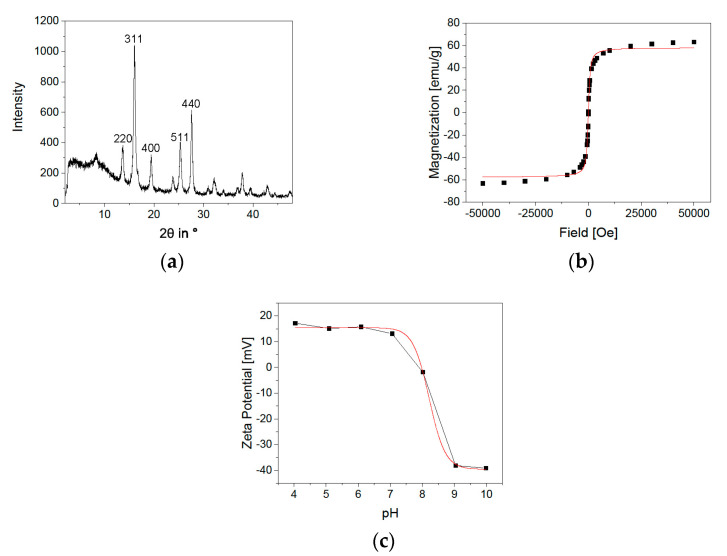
(**a**) XRD measurement of BIONs, (**b**) SQUID analysis of BIONs at 300 K with LangevinMod fit and (**c**) Zeta potential of BIONs at pH values from 4 to 10 with a Boltzmann fit.

With an average diameter of 9.93 nm, the results are comparable to previous measurements of BIONs [32]. The particle size distribution of BIONs synthesized by co-precipitation lies between 6 and 14 nm [32,33]. In addition to TEM measurements, the particle size was determined with XRD analysis. Here, the magnetic particles show an average diameter of 9.2 nm ± 0.43 nm, with a deviation of 0.73 nm, similar to the TEM measurements (Figure 3a, Table S1). BET measurements have shown a specific surface area of 115 ± 0.25 m^2^/g (Figure S1). This value is slightly higher compared to preceding determinations of the specific surface area of BIONs of around 80.0 m^2^/g [32].

In addition to the particle size, the crystal structure composition and the particles’ magnetic behavior play an important role in developing a magnetic drug delivery system as these parameters influence the controllability by an external magnetic field [33,34]. Examination of the crystal structure with XRD analysis shows the typical reflections ((220), (311), (400), (511), and (440)) for magnetite with its cubic structure (Figure 3a) [35]. SQUID analysis of the BIONS (Figure 3b, Table S2) displays the typical sigmoidal curve of superparamagnetic nanoparticles in a magnetic field [36]. There is no hysteresis and no remanence at 0 Oe. The maximal reached magnetization is 63.2 emu/g. The modified Langevin fit is not describing the ideal curve shape of a superparamagnetic substance [37]. The slopes at high magnetic fields indicate the existence of paramagnetic material within the sample. This can occur due to free paramagnetic iron ions, water residues, or oxygen [38,39,40]. Figure 3c shows the zeta potential of BIONs at different pH values from pH 4 to pH 10. The IEP of 7.98 is determined by a Boltzmann fit [41]. This lies between the value of comparable measurements of magnetite with an IEP of 7.0 and the IEP of BIONs that were oxidized by air at 8.4 [32]. In summary, the determined particle size is near the ideal size between 10 and 100 nm, essential for a prolonged blood circulation time. The high magnetizability offers good manageability by a magnetic field with the necessary superparamagnetic behavior for drug delivery [13,42].

### 2.2. Performance of BIONs as Carrier Material for Antimicrobial Peptides

Adsorption and release experiments are performed with LL III under different conditions to examine the BIONs as drug delivery system for short cationic peptides. Since the buffer composition and the pH influence the binding states of short peptides to the BION surface, these parameters are analyzed in the binding experiments [43].

**Figure 4:**
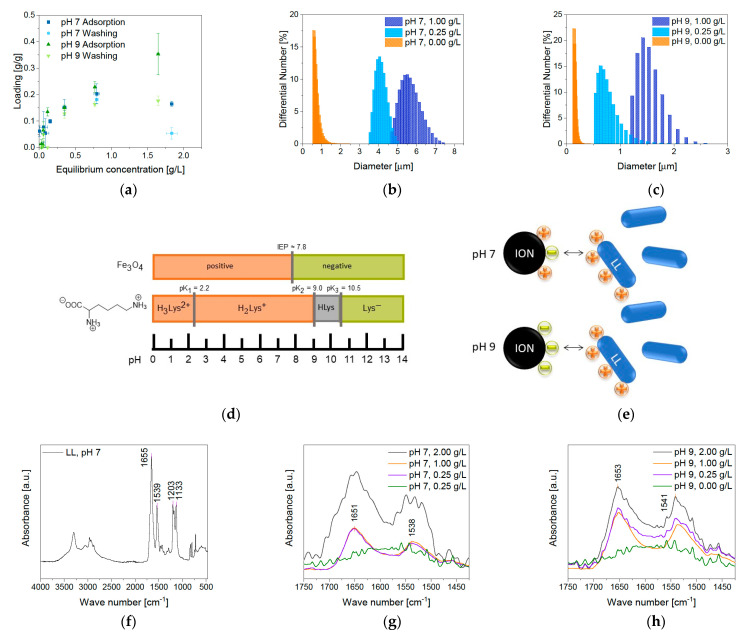
(**a**) Adsorption isotherms of LL at pH 7 and 9 in water and 1 g/L of BIONs, (**b**) hydrodynamic diameters after LL adsorption at pH 7 and (**c**) pH 9 in water, (**d**) illustration of the BION and lysine charge in dependence on the pH. Reprinted with permission from J. Phys. Chem. C 2015, 119, 40, 23032–23041. Copyright 2015 American Chemical Society [22,45], (**e**) interaction of BIONs with lasioglossin at pH 7 (top) and pH 8 (bottom) and IR spectra of (**f**) lasioglossin at pH 7, (**g**) MNP@LL complexes at pH 7 and (**h**) at pH 9 with different starting peptide concentrations.

The BION surface charge (IEP 7.98) at pH 7 in water is expected to be heterogeneous, predominantly positive (Figure 3c). However, small negatively charged domains on the particles can be expected at this pH [44]. On the other hand, we observe a mainly negative charged surface at pH 9 (Figure 4d). This significantly influences the ionic interaction with the peptide LL to the BION surface (Figure 4e). The five lysine groups that generate a positive charge up to pH 9 mainly influence the peptide loading (Figure 4d) [45]. Therefore, they give the peptide a cationic character. Hence, LL is anticipated to have a stronger ionic interaction with the negatively charged BIONs at pH 9 than at pH 7 with the prevalently positive charged surface [45]. The pH-dependent binding behavior of LL to BIONs is presented in Figure 4a (Figure S2, Table S3). As estimated, the highest loading of 0.35 g/g of the BIONs in water is reached at pH 9 (equilibrium concentration: 1.64 g/L), where the particles are negatively, and LL is positively charged. At pH 7, LL shows a maximum binding capacity of 0.20 g/g to BIONs. For both experiments, one washing step leads to a high decrease in loading, so the final loading of BIONs at pH 9 and pH 7 is 0.18 g/g. The average loss of 37% of LL can be explained by the non-covalent reversible binding of the peptide to the iron oxide surface due to electrostatic interactions. The change of the supernatant during the washing step leads to a new equilibrium concentration with a lower loading, since weakly bound LL is removed. The maximal reached drug loading after one washing step in both experiments is 15.2% (Equation (S3)). Other post-loaded nanocarriers where the interaction is also based on hydrophobic, electrostatic interactions, π-π stacking, or hydrogen bonding show drug loading between 11.8% and 68.1% depending on the drug and the material [46]. For iron-based systems, Qu et al. achieved a loading of 9.8% to 11.8% of 10-Hydroxycamptothecin on polyethylene glycol-chitosan coated IONs [47]. In comparison, Luo et al. were able to prepare mesoporous magnetic colloidal nanocrystal clusters that showed a loading capacity of 35% for paclitaxel [48].

The peptide and the BION@LL complex are analyzed with IR for independent validation of the BION LL interaction. LL shows spectroscopy bands between 3000–2800 cm^−1^, 1655–1650 cm^−1,^ and 1542–1539 cm^−1^ due to backbone vibrations. The ones at 3000–2800 cm^−1^ are caused by stretching vibrations of the C–H groups. The band at 1655 cm^−1^ can be affiliated to stretching vibrations of C=O groups and the one at 1539 cm^−1^ to bending vibrations of N–H groups. Additionally, bands at 3400–3300 cm^−1^ are visible in consequence of N–H stretching vibrations and at 3550–3200 cm^−1^ due to the OH group’s stretching vibrations in threonine. Furthermore, LL has characteristic bands at 1203 cm^−1^ and at 1133 cm^-1^ due to C-N stretching of amines and the stretching vibrations of the C–O groups, respectively (Figure 4f) [49,50]. Examining the BION@LL complexes with IR spectroscopy shows the characteristic double bands of LL at 1654 and 1539 cm^−1^ additional to the magnetite peak at ~572 cm^−1^ [51,52] (Figure 4g,h), which proves the formation of the BION peptide complex. Due to inhomogeneities of the sample preparation, the integrals of the bands cannot be used quantitatively. Still, they give a qualitative hint that more LL loading leads to higher characteristic double bands. Further, measurements of the hydrodynamic diameter have shown a significant influence of the presence of LL on particle agglomeration. In different media, the BIONs form different agglomerates. The hydrodynamic diameters are larger than the diameters determined via TEM measurements (Section 2.1). For both aqueous conditions, higher LL concentration led to bigger hydrodynamic diameters and broader particle size distribution. LL can allegedly act as a binder agent due to the multiple cationic functional groups. This effect explains the larger agglomerates along with an increased peptide loading. The starting peptide concentration of 0.25 g/L LL led to agglomerates larger than 4 µm. In contrast, at pH 9 smaller agglomerates were formed, so at 0.25 g/L peptide the hydrodynamic diameter is between 0.50 and 1.70 µm (Figure 4b,c). Other iron post-loaded carriers show sizes between 100 and 600 nm [46,47,48]. In general, the ideal size for a nanoparticle-based drug delivery system is between 10 and 100 nm to achieve long blood circulation times and make cellular uptake possible [18].

**Figure 5:**
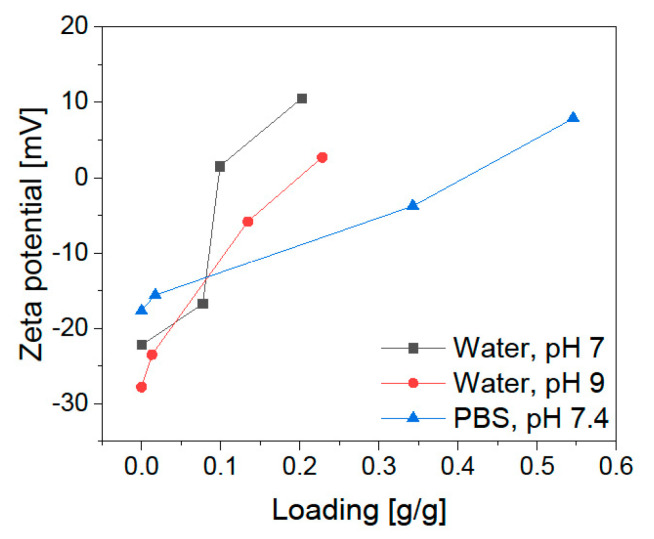
Zeta potential after adsorption of LL to 1 g/L of BIONs at different loadings under the three different conditions.

The amount of LL loading shows an effect on the stability of the colloidal dispersions (Figure 5). With a higher peptide amount the zeta potential is increasing. Low amounts of LL (0 g/L and 0.25 g/L) led to a zeta potential between−10 and−30 mV, so this system shows incipient instability. Higher peptide starting concentrations increase the potentials up to a range of −10 and +10 mV that are characteristic for the formation of agglomerates [53]. This trend corresponds to the measurements of the hydrodynamic diameter.

**Figure 6:**
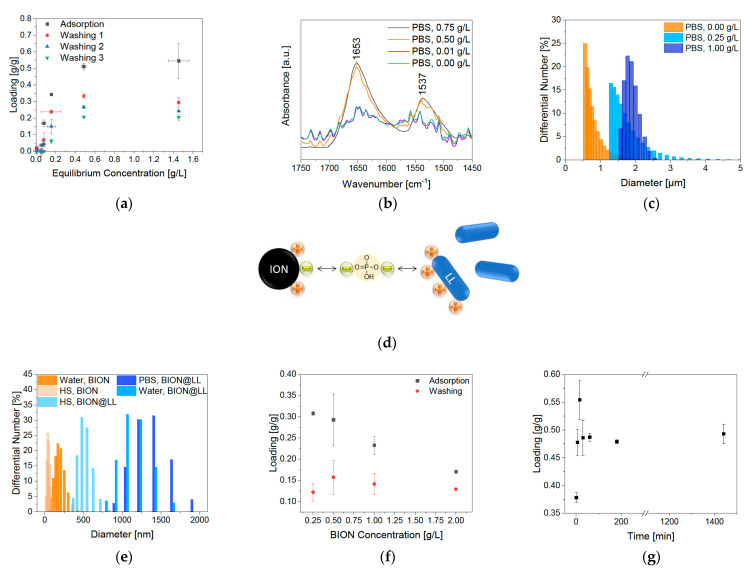
(**a**) Adsorption isotherms of LL at pH 7.4 in 50 mM PBS buffer and 1 g/L of BIONs, (**b**) FTIR spectra of adsorbed LL on BIONs, (**c**) hydrodynamic diameters after LL adsorption at pH 7 in 50 mM PBS buffer, (**d**) illustration of the BION LL interaction in dependence with PBS buffer (**e**). Agglomeration behavior of BIONs and BION@LL in water, human serum (HS) and PBS buffer, (**f**) binding kinetic of 1 g/L LL at pH 7.4 in 50 mM PBS and 1 g/L of BIONs, and (**g**) adsorption of 0.5 g/L LL at pH 7.4 in 50 mM PBS at different BION concentrations.

The influence of the buffer on the system is analyzed by interaction studies in comparable physiological conditions of 50 mM PBS buffer at pH 7.4. This favors the adsorption of LL up to a loading of 0.55 g/g (LL equilibrium concentration 0.46 g/L), while in water the highest loading was 0.35 g/g (Figure 6a). Different effects hereby play a role: (1) the buffer effect completely stabilizes the pH during the adsorption, which could have a positive impact; (2) the negative charge of phosphate can act as a linker between the positive BION surface and the cationic peptide (Figure 6d) and (3) the increase in ionic strength could positively influence the adsorption. Phosphate anions belong to a group that adsorbs by inner-sphere complexation and respond to increasing salt concentration (NaCl) with higher adsorption to metal oxides [54]. Point (2) can be underlined by the zeta potential measurements in Figure 5. Higher peptide loading in PBS does not lead to a significantly increased positive potential. The potential for the higher load is in the same range as the reference sample in water at pH 7. This shows that the phosphate anions affect the surface charge and shield the positive charge of the cationic lasioglossin. Again, washing led to a strong decrease in LL loading. The highest loading after one washing step lowered to 0.23 g/g, due to the new equilibrium adjustment. The determined drug loading is therefore 22.7%. In general, a loss of 41.9% was measured through one washing step.

Further washing steps lead to a decrease in the peptide loading of 28.6% per washing step. More detailed data can be found in the SI in Table S4. Once more, the increase in LL loading was observed in higher characteristic IR peaks (Figure 6b). As anticipated, the behavior of the hydrodynamic diameter and the zeta potential is comparable to the results in water (Figure 6c). The hydrodynamic diameter measurements show that the agglomeration of BIONs and the peptide BION complexes depend on the medium and the lasioglossin loading. Therefore, further experiments are conducted in human serum (HS) to analyze the behavior of BIONs in a more realistic drug delivery environment. HS contains various substances, such as many electrolytes, proteins and peptides, small organic molecules, and nutrients, that can influence the aggregation behavior of BIONs [55]. Furthermore, the viscosity of HS is significantly higher with an average value between 1.10–1.30 mPa·s at 37°C compared to water (0.69 mPa·s) [56]. The viscosity can especially influence the colloidal stability of particles [57]. The experiments were carried out with a concentration of 0.5 g/L BIONs because at these conditions the BION agglomerates are not overlayed by the signals of HS (Figure S3). Experiments with 0.5 g/L BIONs show a lower hydrodynamic diameter in HS (79.1 nm) compared to water at pH 7 (243 nm) (Figure 6e). For BION@LL complexes, this effect is more distinct: the determined hydrodynamic diameters are 1302 nm in PBS, 1070 nm in water, and 470 nm in HS. Therefore, in HS, the particles show diameters in the nanoscale <1000 nm and are better comparable with sizes of other iron-based drug carriers [46,47,48]. Still, the size is not in the ideal range of 10–100 nm. Though the media’s viscosity seems to influence the agglomeration behavior strongly, it can be assumed that blood, with even higher viscosity of around 4 mPa·s, leads to even smaller agglomerates [58]. 

Lower BION concentrations led to an increase in LL loading, while after one washing step all loadings are comparable (Figure 6e). This effect can probably be ascribed to diffusion effects, so LL binds in lower amounts to denser floating agglomerates and particle bulks because it must diffuse into the pores. In contrast, it can bind more efficiently if more space is available around each agglomerate (geometrical heterogeneity). After washing, the loading is comparable for all BION concentrations. For that reason, on every particle an equal amount of binding sites (chemical homogeneity) seem to interact with the peptide. Research in the field of adsorption of inorganic compounds to char has already shown that the chemical nature of the adsorbent material influences the binding more than the geometrical heterogeneity through pores [59].

Furthermore, the binding kinetics in PBS buffer play an important role (Figure 6f). It is determined that significantly rapid adsorption occurs within the first five minutes. After 30 min, the equilibrium of 0.49 g/g LL is reached, not changing in the next 24 h.

### 2.3. Desorption of the Bound Peptides from BIONs

**Figure 7:**
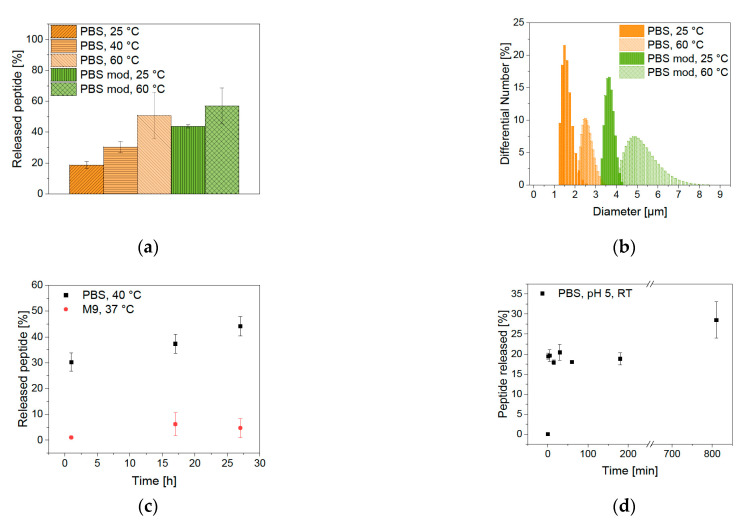
(**a**) Desorption of LL from BIONs at different conditions after one hour, (**b**) agglomeration behavior after one hour of elution at different conditions, (**c**) desorption depending on the time, and (**d**) kinetic of the desorption process of LL in 50 mM PBS, pH 5, and room temperature.

Elution experiments are performed for the analysis of the ability of drug release from the BION peptide complex. When magnetite permeates the cell wall of microorganisms, such as bacteria, an oxidative stress reaction is induced, leading to a pH shift to lower pH values in the cell [60,61]. Furthermore, different compartments of the cell have varying pH values. Endosome and lysosome have an especially low pH of 5 [20]. The impact of a pH shift is tested with elution conditions of PBS buffer at pH 5. Magnetic hyperthermia is a method in which the temperature can be increased up to 40–45 °C by the application of an alternating magnetic field to BIONs [62,63,64], though it is important to note that the agglomeration behavior of the BIONs can have a negative effect on its hyperthermia properties [65]. The desorption is analyzed under possible hyperthermia conditions at a temperature of 40 °C. The temperature sensitivity of the binding is also tested under extreme conditions of 60 °C. Figure 7a shows the effect of the different elution conditions after one hour of incubation. The pH shift from pH 7.4 to pH 5 led only to an elution of 19% LL. Considering that the new equilibrium adjustment plays a role, the pH shift does not influence the elution notably. At pH 5, the surface of the BIONs is predominantly positively charged and can repel the cationic peptide, but the buffer effect of PBS seems to counteract. The additional increase in temperature leads to higher desorption. After one hour at 40 °C 30%, and at 60 °C 51% of LL were desorbed. The peptide binding is therefore temperature-sensitive and the effect of hyperthermia can control elution. The influence of time on the desorption process has been tested by binding kinetics at room temperature in the PBS buffer. Even after 3 h, the elution equilibrium did not change. Only after 13.5 h, 30% of LL have been desorbed (Figure 7d). A temperature of 40 °C leads to elution of 30% after one hour and 44% after 27 h (Figure 7c). The most significant part of the elution takes place in the first minutes, subsequently it is only rising mildly over more extended periods.

The binding properties are analyzed by variation of the sodium chloride amount (0.68 M in PBS buffer, 1.00 M in modified PBS buffer). Higher salt concentration led to elution of 44% at room temperature that is more than twice as much compared with the unmodified PBS buffer. Moreover, a higher temperature of 60 °C led to the highest elution of 57%. This shows that the interaction of LL with the BIONs is electrostatic and non-covalent. Measurements of the hydrodynamic diameter show higher agglomeration after elution with higher salt concentration and higher temperature (Figure 7b). In general, agglomeration can increase the heating efficiency of BIONs but makes it difficult to control the local heating at the target side [66]. For antimicrobial tests in Section 2.4. an M9 medium is used. Analysis of the LL desorption in this medium shows only an elution of 4.7% after 27 h (Figure 7c, Figure S4). Indeed, the full desorption of the peptide from the BIONs is not necessary if it still shows antimicrobial activity while being bound to the particles.

Furthermore, the stability in lysosomal fluid needs to be annotated. Milosevic et al. demonstrated that IONs dissolve in artificial lysosomal fluid within 24 h while forming free iron ions [67]. This iron transfer leads to the degradation and recycling of IONs into ferritin storage [68]. This dissolution could lead to LL release during endocytosis.

### 2.4. Antimicrobial Behavior

**Figure 8:**
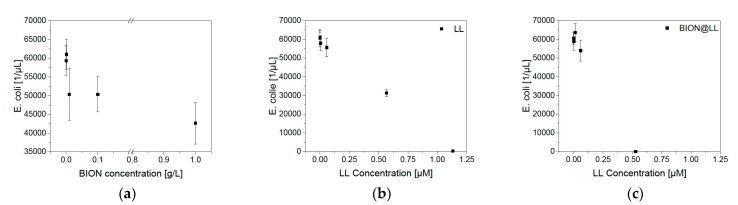
Growth of *E. coli* (BL21 (RH)_4_GFP expressing) in M9 medium under different (**a**) BION concentrations, (**b**) LL concentrations, and (**c**) amounts of the MNP@LL complex. Analysis with microscopy.

LL belongs to the group of antimicrobial peptides (AMPs) and shows antimicrobial activity against various bacteria. For example, for *E. coli* LL-III has a minimum inhibitory concentration (MIC) of 1.4–3.7 μM [25,69]. Furthermore, Zaccharia et al. are reporting a slightly lower MIC of 7.5 μM if LL-III is bound covalently to ureido-pyrimidinone antimicrobial biomaterial [69]. Other short AMPs with many lysines like SYM11KK (KKFPWWWPFKK) or L_9_K_6_ (LKLLKKLLKKLLKLL) show MIC at comparable or slightly higher concentrations of 15 μM and 3.7 μM against *E. coli* [69,70,71]. Various studies of the antimicrobial activity of IONs have already been made. In general, BIONs have an antimicrobial activity at very high concentrations (>50 µM), which can be modified through the surface charge and addition of functional groups. Previous studies have shown that the decrease in BIONs’ size can lead to lower cell growth of *P. aeroginosa* [72,73,74]. The growth rate of green fluorescent protein (GFP) expressing *E. coli* (BL 21 resistant against *ampicillin*) was tested to compare the antimicrobial behavior of LL to the BION@LL complex (Figure 8a). First, the influence of BIONs on cell growth was analyzed. Microscopic cell counting showed that in a 1.00 mg/L BION solution, the growth is comparable to experiments with no presence of BIONs. As expected, higher BION concentrations led to a decrease in *E. coli* growth, but not to full inhibition. The curve shows a negative exponential shape with the smallest colony count for 1.00 g/L BIONs. The antimicrobial peptide LL leads to complete inhibition with a concentration of 1.13 μM, while at lower concentrations a negative linear coherence was observed (Figure 8b). It has to be emphasized that in the Neubauer chamber only fluorescent colonies could be counted and the expression of GFP could be influenced under strong antimicrobial conditions. This could explain the slightly higher MIC compared to the literature discussed above [25,69]. Analysis of the toxic effects of the BION@LL complex shows less bacterial growth with comparable LL concentrations (Figure 8c). Already 0.53 μM LL on the particles lead to complete inhibition, while the peptide only showed less *E. coli* growth. Therefore, the MIC is lower than of bound LL to ureido-pyrimidinone material (7.5 µM) [69]. This can be explained by a better exposure of the peptide and the drug being bound more tightly to the *E. coli* because BIONs can interact with bacteria [75]. Another possibility is the combination of a slight antimicrobial effect of BIONs and the antimicrobial effect of LL which might lead to earlier inhibition. The peptide is fully active while being bound to the particles. Further information and pictures of the *E. coli* in the Neubauer chamber can be found in the Supporting Information in Figures S5–S8.

**Figure 9:**
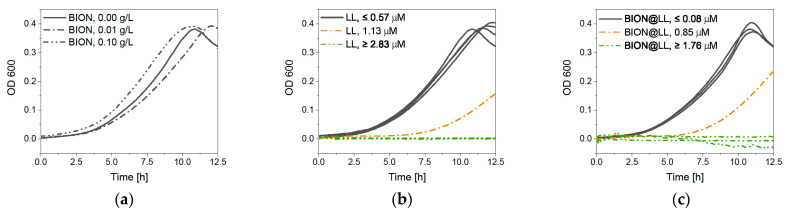
OD_600_ measurements of *E. coli* (BL21 (RH)_4_GFP expressing) in M9 medium under different (**a**) BION concentrations, (**b**) LL concentrations, and (**c**) amounts of the MNP@LL complex. The concentration in the diagram of MNP@LL describes to the LL concentration.

Growth studies with optical density (OD_600_) measurements give comparable results, showing that MNP concentrations up to 0.10 g/L do not negatively influence bacterial growth (Figure 9a). LL leads to less growth at a concentration of 1.13 μM and full inhibition at 2.83 μM or higher (Figure 9b). The BION peptide complexes show a slower growth rate at 0.85 μM of LL and full inhibition at 1.76 μM and higher ones (Figure 9c). The slight differences between these two measurement methods can be ascribed to more intense mixing of the culture in an overhead shaker before microscopy compared to linear shaking of the 96-well plate. Furthermore, for OD_600_ measurements, the ability of GFP expression does not play a role in detection. Both experiments show that the antimicrobial behavior of LL is improved by the binding of LL to the BIONs. 

## 3. Materials and Methods

### 3.1. Synthesis of BIONs

The BIONs are synthesized by co-precipitation. We dissolved 28.9 g sodium hydroxide (723 mmol, 10.3 Eq.) in 400 mL of degassed deionized water under nitrogen atmosphere. A solution of 34.6 g FeCl_3_(H_2_O)_6_ (128 mmol, 1.82 Eq.) and 14.0 g FeCl_2_(H_2_O)_4_ (70.4 mmol, 1.00 Eq., Sigma Aldrich, Merck KGaA, St. Louis, MO, USA), in 160 mL degassed deionized water is slowly added under stirring and temperature control with a water bath at 27 °C. Immediately, a black precipitate forms after the complete addition of all chemicals. The reaction is continued for half an hour under constant conditions. The resulting particles are washed with degassed deionized water (15×) by magnetic decantation until a conductivity of less than 200 μS/cm is reached. The particles are stored in degassed deionized water under nitrogen atmosphere at 4 °C.

### 3.2. Characterization

The magnetic susceptibility is analyzed with a superconducting quantum interference device (SQUID) Quantum Design MPMS XL-7 at 300 K. The magnetic field varied from −50 kOe to +50 kOe. Before the analysis, the particles are lyophilized and glued into a small tube. Transmission electron microscopy (TEM) is performed with the JEM 1400 Plus microscope from JEOL. The sample (10 µL) dispersed in chloroform is dried on a carbon-coated copper grid that has been prepared via glow discharge for sample preparation. The recorded images are subsequently evaluated by using ImageJ software. For this, 30 particles are measured in at least three different areas. Furthermore, powder X-ray diffraction (XRD) is executed with a STOE Stadi-P diffractometer with a molybdenum source (λ = 0.7093 Å) and freeze-dried IONs. The determination of the zeta potential and the hydrodynamic diameter by DLS is performed with a Beckman Coulter Delsa Nano C Particle Analyzer of a 1 g/L ION solution. Each sample is measured in triplicate (cuvette, 10 mm length). The Fourier-transform infrared spectra (FTIR) measurement is carried out with a Bruker ALPHA II spectrometer and the matching platinum attenuated total reflection module, 64 scans per sample. After the measurements have been performed, a concave rubber band method is used to subtract a background in the software OPUS 8.1. For absorbance analysis, the photometer Tecan Infinite M200 PRO Series is used with the evaluation software Magellan. Analysis of the OD_600_ value is implemented with an Eppendorf BioSpectrometer. The BET surface evaluation took place with a Gemini VII (Micromeritics) at 77 K and nitrogen atmosphere, while the volume was determined with helium. Microscopic images are made with an AXIO Observer from Zeiss with an Axiocam 506 mono. For fluorescence analysis, a 475 nm LED is used with 20% intensity for 500 ms, while pictures of BIONs are generated with a transmitted light lamp in brightfield with 12% intensity for 500 ms.

### 3.3. Binding of Peptides to the Nanoparticles

*pH and peptide concentration:* Before conducting the adsorption experiments, the absorbance of LL III solutions, obtained from Gen-Script (Netherlands), with different concentrations are measured in triplicates. We mixed 250 µL of peptide solutions with different concentrations and pH values with 250 µL of a 2 g/L BION stock solution to generate a BION concentration of 1 g/L (LL end concentration: 2.00 g/L, 1.00 g/L, 0.50 g/L, 0.25 g/L, 0.10 g/L, 0.05 g/L, 25.0 mg/L, and 0.00 g/L). Prior, the BIONs are ultrasonicated (20%, 7 min, 10 s on, 15 s off). The experiments are performed in Millipore water (water type 1) at pH 7 and pH 9. The samples are incubated for one hour at 23 °C (1000 rpm) to induce peptide binding. After magnetic decantation, 100 µL of the supernatant are photometrically analyzed at 280 nm. For washing, the particles are resuspended in water at the same pH value and incubated for ten minutes (23 °C, 1000 rpm). Afterwards, the supernatant of the washing step is also analyzed. 

*Buffer and peptide concentration:* The LL stock solution and further dilutions are prepared with 100 mM PBS buffer (3.20 g NaCl, 80.0 mg KCl, 576 mg NaH_2_PO_4_, 96.0 mg KH_2_PO_4_ in 40 mL Millipore water) and afterwards mixed with the BION stock solution in Millipore water at pH 7.4 to generate an overall PBS concentration of 50 mM. Adsorption experiments are performed as described above. The BIONs are washed three times, and the supernatant is analyzed at 230 nm and 280 nm. All further experiments are performed with BION@LL complexes formed in PBS buffer. 

*Particle concentration:* BION solutions of different concentrations in Millipore water at pH 7 (resulting particle concentration: 2.00 g/L, 1.00 g/L, 0.50 g/L, and 0.25 g/L) are mixed with a 2 g/L LL solution in 100 mM PBS buffer. Adsorption experiments are performed as described above and the supernatant is analyzed at 280 nm.

*Time:* 575 µL of 2 g/L LL solution in 100 mM PBS buffer at pH 7 are combined with 575 µL of 2 g/L BION stock solution at pH 7 and incubated at 23 °C (1000 rpm). After 1 min, 5 min, 15 min, 30 min, 60 min, 180 min, and 24 h, each 120 µL of suspension are removed, magnetically decanted, and the supernatant is analyzed photometrically at 280 nm.

### 3.4. Agglomeration Behavior in HS

Suspensions of 0.5 g/L of BIONs and BION@LL (1 g/L LL starting concentration for adsorption in PBS) are ultrasonicated in an ultrasonic bath for 10 min. Through magnetic decantation, the supernatant is exchanged with PBS 50 mM pH 7.4, water pH 7.0, or heat-inactivated AB HS of a human male (Sigma Aldrich). Before the DLS measurement, the particle suspensions are brought to a temperature of 37 °C and then analyzed with the Zetasizer at a measurement temperature of 37 °C.

### 3.5. Elution of Lasioglossin from the Nanoparticles

*Salt concentration and temperature*: The washed lasioglossin-magnetite particles are brought to the same concentration with a 50 mM PBS buffer (1.20 g NaCl, 30.0 mg KCl, 216 mg NaH_2_PO_4_, 36.0 mg KH_2_PO_4_ in 30 mL Millipore water) and a modified PBS buffer (1.75 g NaCl, 30.0 mg KCl, 216 mg NaH_2_PO_4_, 36.0 mg KH_2_PO_4_ in 30 mL Millipore water) at pH 5. After one hour, 17 h or 27 h of incubation (1000 rpm) at 25 °C, 40 °C or 60 °C, and magnetic decantation, the supernatant’s LL concentration is analyzed at 230 nm.

*Time:* After one washing step, the BION@LL complexes are mixed with 50 mM PBS buffer and incubated at 23 °C (1000 rpm). After 1 min, 5 min, 15 min, 30 min, 60 min, 180 min, and 13.5 h 120 µl of each suspension are removed, magnetically decanted and then the supernatant is analyzed photometrically at 230 nm.

### 3.6. Growth Studies

A 1 g/L LL solution in 50 mM PBS buffer (pH 7.4) is sterilized by filtration (0.22 µm filter). Furthermore, a 10 g/L BION solution is prepared in 50 mM PBS buffer. For the BION@LL complex, an adsorption experiment including one washing step is performed in 1 g/L LL solution and 1 g/L BIONs according to Section 2.4. Before, the tests solutions with different concentrations of LL (1.00 g/L, 0.50 g/L, 0.25g/L, 0.10 g/L, 0.05 g/L, 0.02 g/L, 0.01 g/L, 0.001 g/L, and 0.0001 g/L) and BIONs (10.0 g/L, 1.00 g/L, 0.10 g/L), are prepared by dilution of the stock solution with 50 mM PBS buffer in sterile LoBind reaction tubes (Eppendorf). 

*Cell number:* M9 medium is mixed with a 100 g/L ampicillin solution (1:1000) (Table S5). An overnight culture of (RH)_4_-GFP expressing *E. coli* BL21 (DE3) (resistance against ampicillin, incubation at 37 °C) is diluted to OD_600_ 0.01, and 270 µL are mixed with BION and LL and BION@LL solutions in LoBind reaction tubes as described above. As a blank, 30 µL of 50 mM PBS buffer are used. The suspensions are incubated at 37 °C in an overhead shaker for 5 h (500 rpm). After the addition of 3.00 µl of a 0.10 M isopropyl-β-D-thiogalactopyranoside (IPTG) solution, the samples are further incubated for 18 h. After dilution of 10^−2^, they are microscopical analyzed in a Neubauer chamber improved. Through the fluorescence of GFP, the bacteria can be differentiated to the BION agglomerates and counted.

*Cell density:* 180 µL of the same *E. coli* dilution are mixed with 20 µL of the BION, LL, and MNP@LL solutions in a sterile 96-well plate. For the blank, 20 µL of PBS buffer are used instead. Furthermore, for each sample, the same amount of probe is mixed with M9 medium to subtract the influence on the OD_600_ from the different amounts of particles and LL. The probes are incubated at 37 °C and measured every 10 min, with a 60 sec linear amplitude, 1 mm frequency, 173.9 rpm, and 25 read operations.

## 4. Conclusions

It is possible to couple LL as a cationic peptide efficiently to the BION surface. The pH and PBS buffer application play a particular role in LL interaction with BIONs and the binding capacity. The absorbance measurements and IR spectroscopy verify the successful drug loading of 22.7% in PBS buffer. The equilibrium loading is already reached after 30 min and is not further influenced by time. The amount of bound LL and the medium highly influence the agglomeration behavior of BIONs and the electrical potential on the surface. In HS, the hydrodynamic diameter is distinctly smaller than in water or PBS. Each washing step leads to a new equilibrium and, therefore, to LL loss due to reversible binding. Experiments with different amounts of BIONs have shown that this equilibrium is independent of the particle concentration.

Desorption of LL from BIONs is possible. While a pH shift is not showing effective desorption, the binding is temperature-sensitive. Incubation time does not significantly affect LL elution. Only long incubation times of 12–27 h lead to higher elution. The hydrodynamic diameter is strongly influenced by increasing salt concentration and higher temperature leading to big agglomerates. In bacterial growth experiments, the BION@LL complexes show higher antimicrobial activity compared to the antimicrobial peptide alone. 

Thus, LL can be bound in high amounts to the BION surface, and the system is especially effective in growth studies; BIONs show agglomeration beyond 100 nm during all tested conditions. This impedes the applicability in the human body. Biocompatible coatings should be analyzed to stabilize the particles and enhance the tolerability of the human system. The washing and desorption processes need to be further analyzed to generate particles usable for controlled drug delivery. 

The experiments have shown that new antimicrobial peptides can be combined with cost-efficient BIONs to generate a new drug delivery system. The adsorption process can be performed quickly and simply due to electrostatic binding, while the antimicrobial activity is not affected by the binding. The excellent combination of LL with IONs could lead to an efficient magnetic drug delivery system for anticancer treatment in the future, even if further optimization steps are needed.

## Data Availability

All data generated or analyzed during this study are included in this published article and its supplementary information files. Further datasets used and/or analyzed during the study are available from the corresponding author on reasonable request.

## Supplementary Materials

The following materials are available online at https://www.mdpi.com/article/10.3390/ph14050405/s1: Figure S1: BET measurement: Adsorbed nitrogen to relative pressure; Figure S2: *E. coli* colony grown in M9 media incubated with different concentrations of BIONs; Figure S3: *E. coli* colony grown in M9 media incubated with different concentrations of LL; Figure S4: *E. coli* colony grown in M9 media incubated with different concentrations of BION@LL; Figure S5: Amount of LL loaded on BIONs; Table S1: Modified Langevin fir for SQUID Analysis; Table S2: BION size calculation with Scherer equation from XRD; Table S3: Components of M9 Medium for 1 L; Table S4: Components for LB agar plates for 500 mL water title; Equation (S1): Boltzmann fit for Zeta Potential Analysis; Equation (S2): Scherer equation; Equation (S3): Drug loading.

## References

[1] Tiwari G., Tiwari R., Bannerjee S.K., Bhati L., Pandey S., Pandey P., Sriwastawa B. (2012). Drug delivery systems: An updated review. Int. J. Pharm. Investig..

[2] Langer R. (1998). Drug delivery and targeting. Nature.

[3] Piktel E., Niemirowicz K., Wątek M., Wollny T., Deptuła P., Bucki R. (2016). Recent insights in nanotechnology-based drugs and formulations designed for effective anti-cancer therapy. J. Nanobiotechnol..

[4] Alvarez-Lorenzo C., Concheiro A. (2013). Smart Materials for Drug Delivery.

[5] Gupta A.K., Gupta M. (2005). Synthesis and surface engineering of iron oxide nanoparticles for biomedical applications. Biomaterials.

[6] Bruschi M.L., Toledo L.D.A.S.D. (2019). Pharmaceutical Applications of Iron-Oxide Magnetic Nanoparticles. Magnetochemistry.

[7] Wu S., Sun A., Zhai F., Wang J., Xu W., Zhang Q., Volinsky A.A. (2011). Fe_3_O_4_ magnetic nanoparticles synthesis from tailings by ultrasonic chemical co-precipitation. Mater. Lett..

[8] Cao S.-W., Zhu Y.-J., Ma M.-Y., Li L., Zhang L. (2008). Hierarchically Nanostructured Mag-netic Hollow Spheres of Fe3O4 and γ-Fe2O3: Preparation and Potential Application in Drug Delivery. J. Phys. Chem. C.

[9] Laurent S., Forge D., Port M., Roch A., Robic C., Elst L.V., Muller R.N. (2008). Magnetic Iron Oxide Nanoparticles: Synthesis, Stabilization, Vectorization, Physicochemical Characterizations, and Biological Applications. Chem. Rev..

[10] Shaghasemi B.S., Virk M.M., Reimhult E. (2017). Optimization of Magneto-thermally Controlled Release Kinetics by Tuning of Magnetoliposome Composition and Structure. Sci. Rep..

[11] Wnorowska U., Fiedoruk K., Piktel E., Prasad S.V., Sulik M., Janion M., Daniluk T., Savage P.B., Bucki R. (2020). Nanoantibiotics containing membrane-active human cathelicidin LL-37 or synthetic ceragenins attached to the surface of magnetic nanoparticles as novel and innovative therapeutic tools: Current status and potential future applications. J. Nanobiotechnol..

[12] Křížek M., Pechoušek J., Tuc;ek J., Šafářová K., Medřík I., Machala L. Iron oxide nanoparticle powders with high surface area. Proceedings of the AIP Conference.

[13] Lu A.-H., Salabas E.-L., Schüth F. (2007). Magnetic Nanoparticles: Synthesis, Protection, Functionalization, and Application. Angew. Chem. Int. Ed..

[14] Yusoff Ahmad H.M., Salimi Midhat N., Jamlos Mohd F. (2018). A review: Synthetic strategy control of magnetite na-noparticles production. Adv. Nano Res..

[15] Wu W., Wu Z., Yu T., Jiang C., Kim W.-S. (2015). Recent progress on magnetic iron oxide nanoparticles: Synthesis, surface functional strategies and biomedical applications. Sci. Technol. Adv. Mater..

[16] Arruebo M., Fernández-Pacheco R., Ibarra M.R., Santamaría J. (2007). Magnetic nanoparticles for drug delivery. Nano Today.

[17] Israel L.L., Galstyan A., Holler E., Ljubimova J.Y. (2020). Magnetic iron oxide nanoparticles for imaging, targeting and treatment of primary and metastatic tumors of the brain. J. Control. Release.

[18] Kievit F.M., Zhang M. (2011). Surface Engineering of Iron Oxide Nanoparticles for Targeted Cancer Therapy. Acc. Chem. Res..

[19] Liechty W.B., Kryscio D.R., Slaughter B.V., Peppas N.A. (2010). Polymers for Drug Delivery Systems. Annu. Rev. Chem. Biomol. Eng..

[20] Sun T., Zhang Y.S., Pang B., Hyun D.C., Yang M., Xia Y. (2014). Engineered Nanoparticles for Drug Delivery in Cancer Therapy. Angew. Chem. Int. Ed..

[21] Schwaminger S.P., Blank-Shim S.A., Scheifele I., Pipich V., Fraga-García P., Berensmeier S. (2019). Design of Interactions Between Nanomaterials and Proteins: A Highly Affine Peptide Tag to Bare Iron Oxide Nanoparticles for Magnetic Protein Separation. Biotechnol. J..

[22] Schwaminger S.P., García P.F., Merck G.K., Bodensteiner F.A., Heissler S., Günther S., Berensmeier S. (2015). Nature of Interactions of Amino Acids with Bare Magnetite Nanoparticles. J. Phys. Chem. C.

[23] Schwaminger S., Blank-Shim S.A., Borkowska-Panek M., Anand P., Fraga-García P., Fink K., Wenzel W., Berensmeier S. (2018). Experimental characterization and simulation of amino acid and peptide interactions with inorganic materials. Eng. Life Sci..

[24] Rawlings A.E., Bramble J.P., Tang A.A.S., Somner L.A., Monnington A.E., Cooke D.J., McPherson M.J., Tomlinson D.C., Staniland S.S. (2015). Phage display selected magnetite interacting Adhirons for shape controlled nanoparticle synthesis. Chem. Sci..

[25] Ceřovský V., Budešínský M., Hovorka O., Cvac;ka J., Voburka Z., Slaninová J., Borovic;ková L., Fuc;ík V., Bednárová L., Votruba I. (2009). Lasioglossins: Three Novel Antimicrobial Peptides from the Venom of the Eusocial BeeLasioglossum laticeps(Hymenoptera: Halictidae). ChemBioChem.

[26] Hancock R.E.W., Sahl H.-G. (2006). Antimicrobial and host-defense peptides as new anti-infective therapeutic strategies. Nat. Biotechnol..

[27] Parisien A., Allain B., Zhang J., Mandeville R., Lan C. (2007). Novel alternatives to antibiotics: Bacteriophages, bacterial cell wall hydrolases, and antimicrobial peptides. J. Appl. Microbiol..

[28] Bahar A.A., Ren D. (2013). Antimicrobial Peptides. Pharmaceuticals.

[29] Aoki W., Ueda M. (2013). Characterization of Antimicrobial Peptides toward the Development of Novel Antibiotics. Pharmaceuticals.

[30] Brogden K.A. (2005). Antimicrobial peptides: Pore formers or metabolic inhibitors in bacteria?. Nat. Rev. Genet..

[31] Mishra B., Basu A., Saravanan R., Xiang L., Yang L.K., Leong S.S.J. (2013). Lasioglossin-III: Antimicrobial characterization and feasibility study for immobilization applications. RSC Adv..

[32] Schwaminger S.P., Bauer D., Fraga-García P., Wagner F.E., Berensmeier S. (2016). Oxidation of magnetite nanoparticles: Impact on surface and crystal properties. CrystEngComm.

[33] Schnell F., Kube M., Berensmeier S., Schwaminger S.P. (2019). Magnetic Recovery of Cellulase from Cellulose Substrates with Bare Iron Oxide Nanoparticles. ChemNanoMat.

[34] Shang L., Nienhaus K., Nienhaus G.U. (2014). Engineered nanoparticles interacting with cells: Size matters. J. Nanobiotechnol..

[35] Dodi G., Hritcu D., Draganescu D., Popa M.I. (2015). Iron oxide nanoparticles for magnetically assisted patterned coatings. J. Magn. Magn. Mater..

[36] Bean C.P., Livingston J.D. (1959). Superparamagnetism. J. Appl. Phys..

[37] Henrard D., Vuong Q.L., Delangre S., Valentini X., Nonclercq D., Gonon M.F., Gossuin Y. (2019). Monitoring of Superparamagnetic Particle Sizes in the Langevin Law Regime. J. Nanomater..

[38] Eisenberg R., Gray H.B. (2008). Preface on Making Oxygen. Inorg. Chem..

[39] Schweser F., Deistung A., Lehr B.W., Reichenbach J.R. (2010). Differentiation between diamagnetic and paramagnetic cerebral lesions based on magnetic susceptibility mapping. Med Phys..

[40] Walker N. (1977). Paramagnetic properties of Fe(II) and Fe(III). J. Chem. Educ..

[41] Hoppe T. (2013). A simplified representation of anisotropic charge distributions within proteins. J. Chem. Phys..

[42] Dobson J. (2008). Magnetic nanoparticles for gene and drug delivery. Int. J. Nanomed..

[43] Schwaminger S.P., Blank-Shim S.A., Scheifele I., Fraga-García P., Berensmeier S. (2017). Peptide binding to metal oxide nanoparticles. Faraday Discuss..

[44] Blank-Shim S.A., Schwaminger S.P., Borkowska-Panek M., Anand P., Yamin P., Fraga-García P., Fink K., Wenzel W., Berensmeier S. (2017). Binding patterns of homo-peptides on bare magnetic nanoparticles: Insights into environmental dependence. Sci. Rep..

[45] Nelson D.L., Lehninger A.L., Cox M.M. (2013). Lehninger Principles of Biochemistry.

[46] Liu Y., Yang G., Jin S., Xu L., Zhao C. (2020). Development of High-Drug-Loading Nanoparticles. ChemPlusChem.

[47] Qu J.-B., Shao H.-H., Jing G.-L., Huang F. (2013). PEG-chitosan-coated iron oxide nanoparticles with high saturated magnetization as carriers of 10-hydroxycamptothecin: Preparation, characterization and cytotoxicity studies. Colloids Surf. B Biointerfaces.

[48] Luo B., Xu S., Luo A., Wang W.-R., Wang S.-L., Guo J., Lin Y., Zhao D.-Y., Wang C.-C. (2011). Mesoporous Biocompatible and Acid-Degradable Magnetic Colloidal Nanocrystal Clusters with Sustainable Stability and High Hydrophobic Drug Loading Capacity. ACS Nano.

[49] Ebrahiminezhad A., Ghasemi Y., Rasoul-Amini S., Barar J., Davaran S. (2012). Impact of Amino-Acid Coating on the Synthesis and Characteristics of Iron-Oxide Nanoparticles (IONs). Bull. Korean Chem. Soc..

[50] Morhardt C., Ketterer B., Heißler S., Franzreb M. (2014). Direct quantification of immobilized enzymes by means of FTIR ATR spectroscopy–A process analytics tool for biotransformations applying non-porous magnetic enzyme carriers. J. Mol. Catal. B Enzym..

[51] Bordbar A.K., Rastegari A.A., Amiri R., Ranjbakhsh E., Abbasi M., Khosropour A.R. (2014). Characterization of Modified Magnetite Nanoparticles for Albumin Immobilization. Biotechnol. Res. Int..

[52] Sigma Aldrich IR Spectrum Table & Chart. https://www.sigmaaldrich.com/technical-documents/articles/biology/ir-spectrum-table.html.

[53] Kumar A., Dixit C.K. (2017). Methods for characterization of nanoparticles. Advances in Nanomedicine for the Delivery of Therapeutic Nucleic Acids.

[54] Almasri D.A., Saleh N.B., Atieh M.A., McKay G., Ahzi S. (2019). Adsorption of phosphate on iron oxide doped halloysite nanotubes. Sci. Rep..

[55] Psychogios N., Hau D.D., Peng J., Guo A.C., Mandal R., Bouatra S., Sinelnikov I., Krishnamurthy R., Eisner R., Gautam B. (2011). The Human Serum Metabolome. PLoS ONE.

[56] Késmárky G., Kenyeres P., Rábai M., Tóth K. (2008). Plasma viscosity: A forgotten variable. Clin. Hemorheol. Microcirc..

[57] Benítez E.I., Genovese D.B., Lozano J.E. (2009). Effect of typical sugars on the viscosity and colloidal stability of apple juice. Food Hydrocoll..

[58] Coppola L., Caserta F., De Lucia D., Guastafierro S., Grassia A., Coppola A., Marfella R., Varricchio M. (2000). Blood viscosity and aging. Arch. Gerontol. Geriatr..

[59] Yakout S.M. (2016). Effect of porosity and surface chemistry on the adsorption-desorption of uranium(VI) from aqueous solution and groundwater. J. Radioanal. Nucl. Chem..

[60] Zhu M.-T., Wang Y., Feng W.-Y., Wang B., Wang M., Ouyang H., Chai Z.-F. (2010). Oxidative Stress and Apoptosis Induced by Iron Oxide Nanoparticles in Cultured Human Umbilical Endothelial Cells. J. Nanosci. Nanotechnol..

[61] Rivolta I., Panariti A., Miserocchi G. (2012). The effect of nanoparticle uptake on cellular behavior: Disrupting or enabling functions?. Nanotechnol. Sci. Appl..

[62] Hauser A.K., Wydra R.J., Stocke N.A., Anderson K.W., Hilt J.Z. (2015). Magnetic nanoparticles and nanocomposites for remote controlled therapies. J. Control. Release.

[63] Saeed M., Ren W., Wu A. (2017). Therapeutic applications of iron oxide based nanoparticles in cancer: Basic concepts and recent advances. Biomater. Sci..

[64] Gomes I.P., Duarte J.A., Maia A.L.C., Rubello D., Townsend D.M., De Barros A.L.B., Leite E.A. (2019). Thermosensitive Nanosystems Associated with Hyperthermia for Cancer Treatment. Pharmaceuticals.

[65] Abu-Bakr A.F., Zubarev A.Y. (2020). Effect of ferromagnetic nanoparticles aggregation on magnetic hyper-thermia. Eur. Phys. J. Spec. Top..

[66] Perigo E.A., Hemery G., Sandre O., Ortega D., Garaio E., Plazaola F., Teran F.J. (2015). Fundamentals and advances in magnetic hyperthermia. Appl. Phys. Rev..

[67] MilosevicAc A., Bourquin J., Burnand D., Lemal P., Crippa F., Monnier C.A., Rodriguez-Lorenzo L., Petri-Fink A., Rothen-Rutishauser B. (2019). Artificial Lysosomal Platform to Study Nanoparticle Long-term Stability. Chim. Int. J. Chem..

[68] Lartigue L., Alloyeau D., Kolosnjaj-Tabi J., Javed Y., Guardia P., Riedinger A., Péchoux C., Pellegrino T., Wilhelm C., Gazeau F. (2013). Biodegradation of Iron Oxide Nanocubes: High-Resolution In Situ Monitoring. ACS Nano.

[69] Zaccaria S., Van Gaal R.C., Riool M., Zaat S.A.J., Dankers P.Y.W. (2018). Antimicrobial peptide modification of biomaterials using supramolecular additives. J. Polym. Sci. Part A: Polym. Chem..

[70] Braunstein A., Papo N., Shai Y. (2004). In Vitro Activity and Potency of an Intravenously Injected Antimicrobial Peptide and Its dl Amino Acid Analog in Mice Infected with Bacteria. Antimicrob. Agents Chemother..

[71] Papo N., Oren Z., Pag U., Sahl H.-G., Shai Y. (2002). The Consequence of Sequence Alteration of an Amphipathic α-Helical Antimicrobial Peptide and Its Diastereomers. J. Biol. Chem..

[72] Chatterjee S., Bandyopadhyay A., Sarkar K. (2011). Effect of iron oxide and gold nanoparticles on bacterial growth leading towards biological application. J. Nanobiotechnol..

[73] Arakha M., Pal S., Samantarrai D., Panigrahi T.K., Mallick B.C., Pramanik K., Mallick B., Jha S. (2015). Antimicrobial activity of iron oxide nanoparticle upon modulation of nanoparticle-bacteria interface. Sci. Rep..

[74] Borcherding J., Baltrusaitis J., Chen H., Stebounova L., Wu C.-M., Rubasinghege G., Mudunkotuwa I.A., Caraballo J.C., Zabner J., Grassian V.H. (2014). Iron oxide nanoparticles induce Pseudomonas aeruginosa growth, induce biofilm formation, and inhibit antimicrobial peptide function. Environ. Sci. Nano.

[75] Thomas J.A., Schnell F., Kaveh-Baghbaderani Y., Berensmeier S., Schwaminger S.P. (2020). Immunomagnetic Separation of Microorganisms with Iron Oxide Nanoparticles. Chemosensors.

