# Supplementary material for: Bare Iron Oxide Nanoparticles as Drug Delivery Carrier for the Short Cationic Peptide Lasioglossin
Source: Pharmaceuticals (Basel). 2021 Apr 24;14(5):405. doi: 10.3390/ph14050405 (PMC8146918; doi:10.3390/ph14050405)
Supplement: Supplementary file 1 [file pharmaceuticals-14-00405-s001.zip › pharmaceuticals-1175094-supplementary.pdf]

pharmaceuticals

MDPI

Supporting Materials

Bare iron oxide nanoparticles as drug delivery carrier for the short cationic peptide lasioglossin

Chiara Turrina [1], Sonja Berensmeier [1] and Sebastian P. Schwaminger [1,*]

[1] Bioseparation Engineering Group, Department of Mechanical Engineering, Technical University of Munich
* Correspondence: s.schwaminger@tum.de

Particle characterization

Table S1: BION Size calculation with the Scherrer equation from XRD data, measured with Mo K_α. The lattice constant is calculated from the Bragg equation.

2θ	16.01	13.72	19.40	Average
Size	9.07 nm	8.71 nm	9.74 nm	9.17 nm

Scherrer equation:

$$L = \frac{0.89 \cdot 0.07093\ nm}{\Delta 2\theta \cdot \cos(\theta)} \qquad \text{(S2)}$$

Table S2: Modified Langevin fit for SQUID Analysis

	y0	xc	C	s
Magnetization	0.13902±0.66446	2.96189±25.77472	58.02718±0.79117	352.84062±22.54824

Boltzmann fit for Zeta Potential Analysis:

$$y=-39.61+(15.46-(-39.61))/(1+(e^{x-8.21)/0.25})) \qquad \text{(S1)}$$

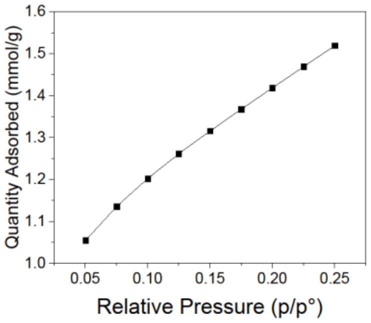

Figure S1: BET measurement: Adsorbed nitrogen to relative pressure.

Drug loading

$$\text{Drug loading} = \frac{m(drug)}{m(drug)+m(nanocarrier)} \qquad \text{(S3)}$$

Figure S2: Exemplary calibration curves for the photometric measurements for the adsorption and release experiments.

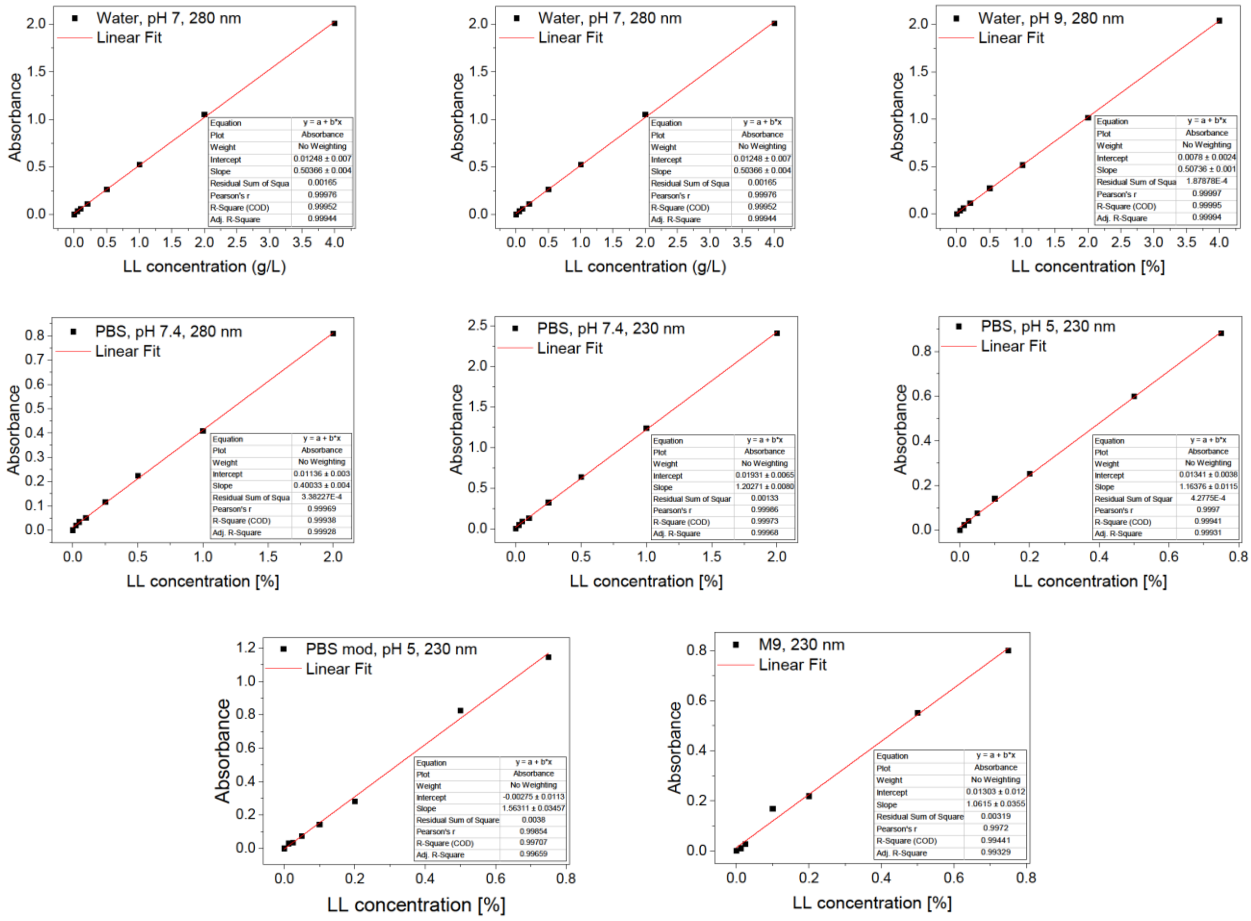

A new calibration curve was generated for each measurement, so these curves are exemplary for all the used conditions.

Table S3: Data of Adsorption and release experiments.

LL Conc.	Adsorption: Water, pH 7, 280 nm			Washing 1: Water, pH 7, 280 nm			Adsorption: Water, pH 9, 280 nm			Washing 1: Water, pH 9, 280 nm		
	Absorbance			Absorbance			Absorbance			Absorbance		
[g/L]	Value 1	Value 2	Value 3	Value 1	Value 2	Value 3	Value 1	Value 2	Value 3	Value 1	Value 2	Value 3
0	0.0727	0.059	0.0773	0.0414	0.0427	0.0414	0.0409	0.0432	0.0414	0.0403	0.0401	0.0403
0.025	0.0558	0.0972	0.127	0.0422	0.0426	0.0815	0.0656	0.0756	0.0555	0.0938	0.0767	0.0593
0.05	0.0839	0.0622	0.0822	0.0532	0.045	0.0452	0.0816	0.0698	0.0819	0.052	0.056	0.0496
0.1	0.107	0.103	0.162	0.0429	0.0478	0.0465	0.062	0.073	0.101	0.0441	0.046	0.0558
0.25	0.163	0.155	0.156	0.0496	0.0499	0.0455	0.113	0.0975	0.114	0.0423	0.0422	0.0465
0.5	0.262	0.274	0.237	0.0478	0.0496	0.0497	0.223	0.224	0.235	0.0501	0.0565	0.071
1	0.505	0.459	0.487	0.0648	0.0574	0.0739	0.452	0.444	0.427	0.0842	0.0744	0.0845
2	1	1.01	1.01	0.124	0.0969	0.11	0.834	0.892	0.93	0.127	0.136	0.15

LL Conc.	Adsorption: PBS, pH 7.4, 280 nm Absorbance			Washing 1: PBS, pH 7.4, 280 nm Absorbance			Adsorption: PBS, pH 7.4, 230 nm Absorbance			Washing 1: PBS, pH 7.4, 230 nm Absorbance		
[g/L]	Value 1	Value 2	Value 3	Value 1	Value 2	Value 3	Value 1	Value 2	Value 3	Value 1	Value 2	Value 3
0	0.0446	0.0429	0.0439	0.0456	0.0456	0.0455	0.082	0.0794	0.0803	0.0809	0.0809	0.0814
2	0.581	0.683	0.648	0.141	0.161	0.171	1.74	2.01	1.93	0.349	0.387	0.418
1	0.261	0.241	0.25	0.123	0.126	0.136	0.688	0.637	0.641	0.293	0.297	0.305
0.5	0.121	0.113	0.12	0.097	0.0943	0.105	0.274	0.263	0.214	0.209	0.194	0.199
0.25	0.0794	0.0849	0.0989	0.0833	0.12	0.0863	0.148	0.17	0.216	0.147	0.194	0.159
0.1	0.0642	0.0873	0.0895	0.0781	0.0855	0.0968	0.118	0.148	0.15	0.131	0.139	0.152
0.05	0.0693	0.0628	0.0921	0.0643	0.073	0.0915	0.119	0.114	0.148	0.114	0.122	0.144
0.025	0.0571	0.0532	0.0593	0.054	0.0601	0.0786	0.102	0.0958	0.104	0.0942	0.0992	0.125

Elution	LL Start Concentration	Absorbance: 1 h			Absorbance: 17 h			Absorbance: 27 h		
230 nm	[g/L]	Value 1	Value 2	Value 3	Value 1	Value 2	Value 3	Value 1	Value 2	Value 3
PBS, pH 5, 25 °C	1	0.18	0.159	0.164						
	0	0.0801	0.0807	0.08						
PBS, pH 5, 40 °C	1	0.167	0.159	0.168	0.171	0.164	0.181	0.189	0.177	0.196
	0	0.085	0.0859	0.0853	0.0842	0.0842	0.0844	0.0861	0.086	0.0861
PBS, pH 5, 60 °C	1	0.33	0.268	0.205						
	0	0.088	0.0879	0.0888						
PBS mod, pH 5, 25 °C	1	0.23	0.221	0.234						
	0	0.088	0.0889	0.0905						
PBS mod, pH 5, 60 °C	1	0.24	0.245	0.22						
	0	0.0905	0.0909	0.0907						
M9, 37 °C	1	0.2	0.199	0.199	0.219	0.214	0.207	0.236	0.232	0.218
	0	0.185	0.189	0.182	0.201	0.215	0.218	0.211	0.211	0.212

Elution kinetic 230 nm	LL Start Concentration [g/L]	1 min	5 min	15 min	30 min	60 min	180 min	810 min
PBS, pH 5, 25 °C	1	0.23	0.226	0.227	0.233	0.225	0.232	0.264
	1	0.24	0.247	0.221	0.228	0.226	0.238	0.343
	1	0.234	0.234	0.229	0.262	0.227	0.222	0.26
	0	0.113	0.112	0.102	0.106	0.102	0.103	0.107

Adsorption kinetic	LL Starting Concentration							
230 nm	[g/L]	1 min	6 min	16 min	31 min	61 min	181 min	1441 min
	1	0.316	0.287	0.259	0.287	0.27	0.275	0.263
PBS, pH 5, 25	1	0.324	0.267	0.229	0.26	0.276	0.278	0.278
°C	1	0.321	0.277	0.244	0.275	0.272	0.277	0.271
	0	0.0453	0.0461	0.0453				

For the calculation of all experiments the blank is substracted from the adsorption or desorption data.

Table S4: Influence of Washing on the peptide loading in PBS buffer.

Starting peptide concentration [g/L]	Equilibrium peptide concentration [g/L]	Loading Adsorption [g/g]	Loading Washing 1 [g/g]	Loss 1 [%]	Loading Washing 2 [g/g]	Loss 2 [%]	Loading Washing 3 [g/g]	Loss 3 [%]
2.00	1.45	0.55	0.29	46.1	0.24	17.7	0.20	15.5
1.00	0.49	0.51	0.33	34.8	0.27	20.2	0.21	22.2
0.50	0.17	0.34	0.24	30.4	0.15	37.7	0.06	58.4

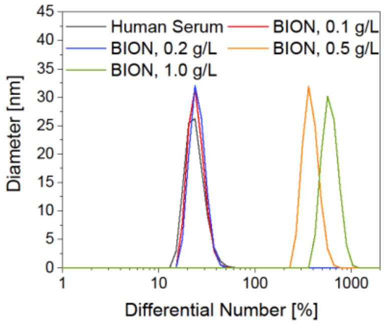

Figure S3: Agglomeration behavior of BIONs in Human Serum depending on the concentration.

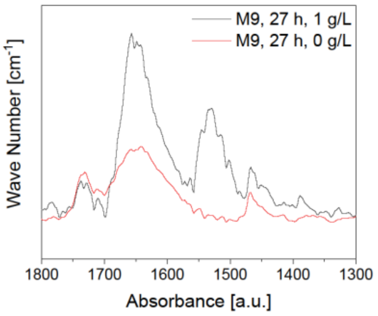

Figure S4: IR spectra of BION@LL and only BIONs after 27 h desorption in M9 medium.

Growth studies

Table S5: Components of M9 Medium for 1 L (867 ml sterile water).

Solution	Composition	Amount of substances	Volume
M9 salt solution (10x)	Na_2HPO_4<br>KH_2PO_4<br>NaCl<br>NH_4Cl	33.7 mM<br>22.0 mM<br>8.55 mM<br>9.35 mM	100 mL
20% glucose solution	Glucose monohydrate	0.40%	20 mL
1 M $MgSO_4$ solution	$MgSO_4$·7 H_2O	1.00 mM	1 mL
1 M $CaCl_2$ solution	$CaCl_2$·2 H_2O	0.30 mM	0.3 mL
1 M mg/mL biotin solution	Biotin	1.00 µg	1 mL
1 M mg/mL thiamin solution	Thiamin	1.00 µg	1 mL
Trace elements solution (100x)	EDTA<br>$FeCl_3$·6 H_2O<br>$ZnCl_2$<br>$CuCl_2$·2 H_2O<br>$CoCl_2$·2 H_2O<br>H_3BO_3<br>$MNCl_2$· 4 H_2O	13.4 mM<br>3.10 mM<br>0.62 mM<br>76.0 µM<br>42.0 µM<br>162 µM<br>8.10 µM	10 mL (1x)

Microscopy

For each measurement, five group squares of biological triplicates are counted.

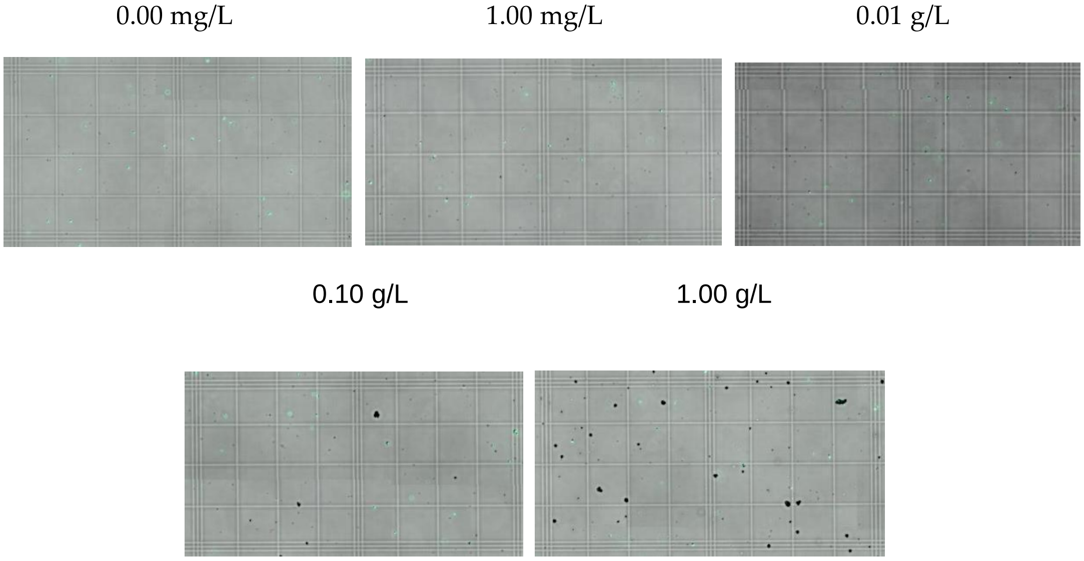

Figure S5: *E. coli* colony grown in M9 media with ampicillin, incubated with different concentrations of BIONs (37 °C, addition of IPTG after 5 h).

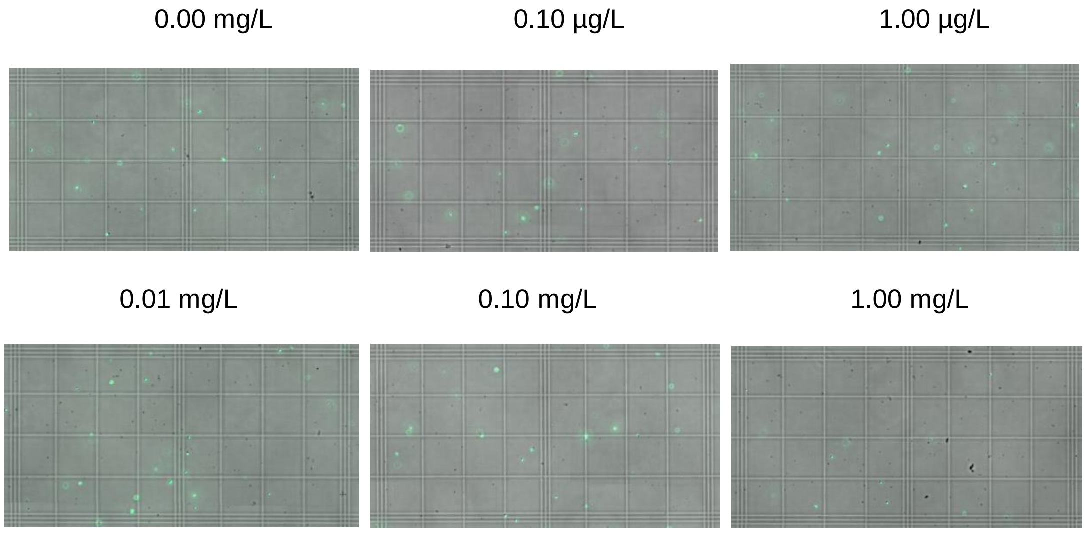

Figure S6: *E. coli* colony grown in M9 media with ampicillin, incubated with different concentrations of LL (37 °C, addition of IPTG after 5 h).

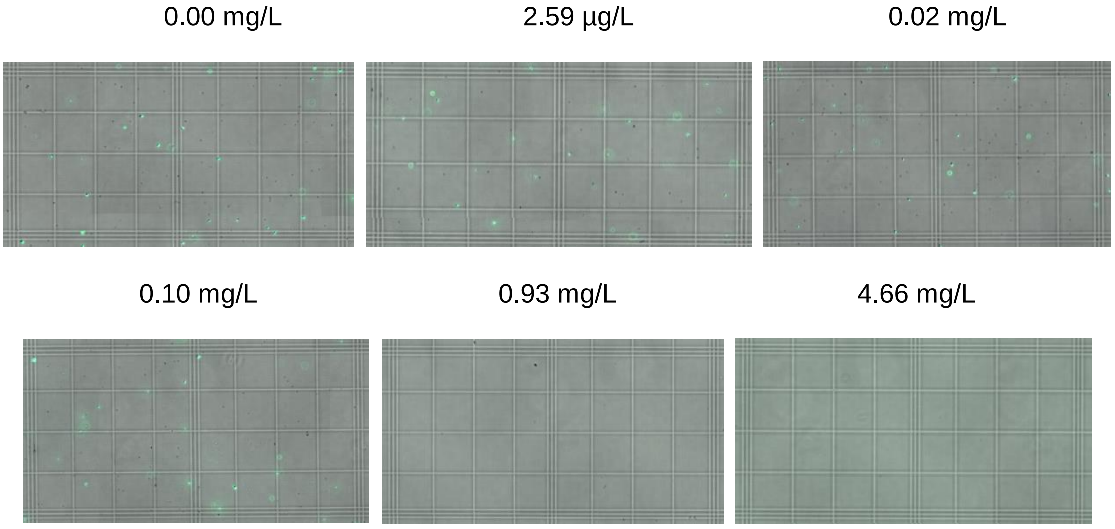

OD_{600}

Figure S7: *E. coli* colony grown in M9 media with ampicillin, incubated with different concentrations of BION@LL (37 °C, addition of IPTG after 5 h).

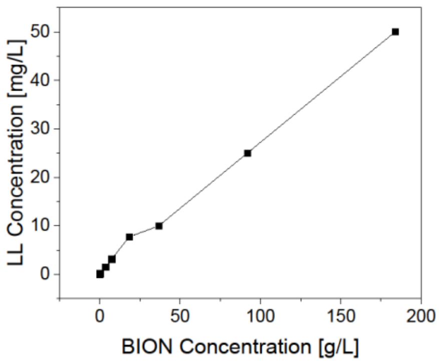

Figure S8: Amount of LL loaded on BIONs.
